# Racial and Ethnic Disparities in the Quality of Diabetes Care in a Nationally Representative Sample

**Published:** 2011-10-15

**Authors:** Patrick Richard, Pierre Kébreau Alexandre, Anthony Lara, Adaeze B. Akamigbo

**Affiliations:** Department of Health Policy, The George Washington University School of Public Health and Health Services; Johns Hopkins Bloomberg School of Public Health, Baltimore, Maryland; Department of Health Policy, The George Washington University School of Public Health and Health Services, Washington, DC; Health Research & Educational Trust, Chicago, Illinois

## Abstract

**Introduction:**

Previous studies have consistently documented that racial/ethnic minority patients with diabetes receive lower quality of care, based on various measures of quality of care and care settings. However, 2 recent studies that used data from Medicare or Veterans Administration beneficiaries have shown improvements in racial/ethnic disparities in the quality of diabetes care. These inconsistencies suggest that additional investigation is needed to provide new information about the relationship between racial/ethnic minority patients and the quality of diabetes care.

**Methods:**

We analyzed 3 years of data (2005-2007) from the Medical Expenditure Panel Survey and used multivariate models that adjusted for sociodemographic characteristics, regional location, insurance status, health behaviors, health status, and comorbidity to examine racial/ethnic disparities in the quality of diabetes care.

**Results:**

We found that Asian patients with diabetes were less likely to have received 2 or more glycated hemoglobin (HbA1c) tests or a foot examination during the past year compared with their white counterparts. Hispanic patients with diabetes were also less likely to have received a foot examination during the past year compared with white patients with diabetes. Conversely, black patients with diabetes were more likely to have received a foot examination during the past year compared with white patients with diabetes. The differences in the quality of diabetes care remained significant even after controlling for socioeconomic status (SES), health insurance status, self-rated health status, comorbid conditions, and lifestyle behavior variables.

**Conclusions:**

Although the link between racial/ethnic minority status and the quality of care for patients with diabetes is not completely understood, our results suggest that factors such as SES, health insurance status, self-rated health status, and other health conditions are potential antecedents of quality of diabetes care.

## Introduction

Although diabetes is a prevalent, debilitating, and costly chronic condition that affects the general population, evidence suggests that racial/ethnic minority groups bear a disproportionate burden of the condition (1-5). Racial/ethnic minority groups have a higher prevalence, worse diabetes outcomes, and higher rates of diabetes-related complications than their white counterparts (1-3,6,7). Previous studies have consistently documented that racial/ethnic minority patients with diabetes receive lower quality of care, based on various measures of quality of care and care settings (8-12). For instance, a report published in 2006 by the Agency for Healthcare Research and Quality showed that racial/ethnic minority groups, including patients who have diabetes, received poorer quality of care in 22 critical measures of quality care compared with whites (13).

However, a recent study that used data from 1997 to 2003 from Medicare beneficiaries in managed care plans has shown that improvements have been made in racial/ethnic disparities in the quality of diabetes care (14). Clinical performance for patients with diabetes improved, and the gap in the quality of diabetes care between whites and blacks narrowed for 7 of the Health Plan Employer Data and Information Set (HEDIS) measures, including glycated hemoglobin (HbA1c) and eye examination. Similarly, a more recent study that used nationally representative data from the Medical Expenditures Panel Survey (MEPS) found no significant differences in the quality of diabetes care between racial/ethnic minority groups and white patients (15).

Additional studies that examined the Medicare and Veterans Administration (VA) populations have suggested that recent investment in public resources to address racial/ethnic inequalities in health and health care may have resulted in the reduction or elimination of racial/ethnic disparities in the quality of diabetes care (14,16,17). The results of these 2 studies are encouraging but cannot be generalized to the US population because of systematic differences between the general population and Medicare or VA beneficiaries. These inconsistencies also suggest that additional investigation is needed to provide new information about the relationship between racial/ethnic minority groups and the quality of diabetes care.

Racial/ethnic differences in the quality of diabetes care may arise from multiple factors and complex interactions between patients, their providers, and the health care systems in which they operate (18). Therefore, we investigated factors that are amenable to policy changes in our models, including socioeconomic status (SES) and health insurance coverage, to determine racial/ethnic differences in the quality of diabetes care. Our objective was to examine racial/ethnic disparities in the quality of care provided to patients with diabetes by using nationally representative data sets.

## Methods

### Sample

We analyzed 3 years of data (2005-2007) from MEPS, a nationally representative survey of health services use, health insurance coverage, medical expenditures, and sources of payment for the US civilian noninstitutionalized population that is cosponsored by the Agency for Healthcare Research and Quality and the National Center for Health Statistics. For this analysis, we used the household component (HC) file of MEPS, which is the core component of the survey that collects data on demographic characteristics, health conditions, self-rated health status, medical services use, access to care, satisfaction with care, health insurance coverage status, and income for each person surveyed.

We pooled 3 years of data to increase the sample size of the study and used a study design that attempted to address previous shortcomings and inconsistencies in the literature. The overlapping design of MEPS allows repeated observations of the same people several times during the year. We combined data from the HC files with the pooled estimation linkage file of MEPS to restrict the analytic sample to unique individuals. By restricting the sample in this way, we were able to compute appropriate standard errors. To construct the analytic sample we used data from the MEPS Diabetes Care Survey, a self-administered questionnaire to adult respondents aged 18 years or older who reported that they had been diagnosed with diabetes by a health care professional. This survey contains a series of questions about diabetes management for 2005, 2006, and 2007, including the number of times respondents reported having had an HbA1c test, the number of times they reported having had their feet checked for sores or irritation, and the last time they reported having had an eye examination during the same period.

The resulting sample was 2,671 people who reported that they had been diagnosed with diabetes by a health care professional during the 3 years combined. We subsequently excluded 182 people, either because they did not respond to the self-administered questionnaire themselves or did not have any office visit during the time of the study. We limited the analytic sample to people who responded to the self-administered questionnaire by themselves, not by their spouse or another proxy, to limit reporting bias and included only patients with diabetes who had at least 1 visit to a health care professional during the past 12 months to capture patient-provider interactions in measuring the quality of care for patients with diabetes. Finally, we excluded an additional 37 people who had missing observations on the different variables used in the analysis. The final analytic sample was 2,452 patients who reported that they had been diagnosed with diabetes by a health care professional and were aged 18 years or older.

### Variables

Consistent with American Diabetes Association guidelines for patients with diabetes, we used 3 binary indicators to measure quality of care for patients with diabetes, which were reporting receipt of the following during the past year: 1) 2 or more HbA1c tests, 2) 1 foot examination, and 3) 1 eye examination. For the HbA1c tests, MEPS asked, "During [survey year], how many times did a doctor, nurse, or other health professional check your blood for glycosylated hemoglobin or 'hemoglobin A-one-C'?" For the foot examination, MEPS asked, "How many times did a health professional check your feet for any sores or irritations?" For the eye examination, MEPS asked, "In which year did you have an eye examination in which your pupils were dilated?"

On the basis of previous research, we controlled for a set of patient characteristics known to be associated with differences in quality of care including age, race/ethnicity, sex, SES, health insurance status, smoking status, obesity status, general health status, comorbid cardiovascular conditions, and regional location (19-21). To assess patients' race/ethnicity, respondents were asked, "Which of these would you say is your main racial or ethnic group?" Response options were non-Hispanic white, non-Hispanic African American, Hispanic, American Indian or Alaska Native, Asian or Pacific Islander, mixed race, or some other single race. From these responses, we constructed 4 categories: non-Hispanic white, black, Hispanic, and Asian. We used the MEPS body mass index (BMI) measure, calculated from respondents' self-reported height and weight, to create an indicator variable for obesity (BMI ≥30 kg/m^2^). Different categories of education and income were used to account for the nonlinearity of the relationship between these 2 variables and the quality of care for patients with diabetes. Education levels were defined as receiving less than a high school degree, high school degree, college degree, or postgraduate degree. Income levels were defined as incomes below 100% of the federal poverty level (FPL), between 100% and 199% of the FPL, between 200% and 400% of the FPL, and above 400% of the FPL. Comorbid cardiovascular conditions included patients who reported being diagnosed with at least 1 of the following conditions: hypertension, angina, mild or coronary heart attack, stroke, or other form of heart disease.

### Statistical analysis

We used logistic regression models to determine the odds of receiving at least 2 HbA1c tests, a foot examination, or an eye examination in the past year. We conducted χ^2^ tests to determine differences in outcomes among the different racial/ethnic minority groups. Significance was set at *P* < .10. Because of the complex survey design of the MEPS HC file, we used special diabetes weights from MEPS to compute robust standard errors of the estimates. Because we pooled data over several years for a subsample of patients with diabetes, we used the balanced repeated replication method of variance estimation to account for the full set of survey stratum and primary sampling units, as recommended by MEPS. Weighted proportions, adjusted odds ratios (AORs), and 95% confidence intervals (CIs) were used to present the results. We used Stata version 11 (StataCorp LP, College Station, Texas) to conduct the analysis.

## Results

More than 68% of respondents were aged 55 years or older, 34% of respondents resided in families with incomes higher than 400% of the FPL, and 31% had some form of public insurance such as Medicaid or Medicare. Approximately 78% of the sample had other comorbid cardiovascular conditions (Table 1).

On average, about 83% of patients reported receiving at least 2 HbA1c tests; 70%, a foot examination; and 61%, an eye examination during the past 12 months. Chi-square tests indicated significant differences between whites and Asians receiving at least 2 HbA1c tests (*P* = .007) and a foot examination (*P* = .002) (Table 2). Hispanic patients were less likely to receive an eye examination during the past year than were white patients (*P* = .005). Conversely, black patients were more likely to receive a foot examination than were white patients (*P* = .009) and less likely to receive an eye examination than were white patients (*P* = .03) during the past year.

Multivariate logistic regression analyses indicated that Asian patients with diabetes were less likely to receive at least 2 HbA1c tests or a foot examination in the past year than were their white counterparts (Table 3). Likewise, Hispanic patients with diabetes were less likely to receive a foot examination in the past 12 months than were white patients with diabetes. Conversely, black patients with diabetes were more likely to receive a foot examination than were white patients with diabetes.

High school graduates were less likely to receive at least 2 HbA1c tests or a foot examination compared with participants who did not graduate from high school. Similarly, patients with diabetes who resided in the Midwest, South, or West were less likely to receive a foot examination than were those who lived in the northeastern part of the country. We also found negative associations between receipt of eye examination and patients who were uninsured or who smoked compared with those who were privately insured or did not smoke, respectively. Patients who resided in families with incomes more than 400% of the FPL, were in fair or poor health, and suffered from comorbid cardiovascular conditions were more likely to report receiving HbA1c tests compared with those who lived in families with incomes below 100% of the FPL, were in excellent or good health, and did not have a comorbid cardiovascular condition. For example, patients with a comorbid cardiovascular condition were 34% more likely to have received 2 or more HbA1c tests than were those who did not.

Patients who resided in families with an income above 400% of the FPL, were publicly insured with either Medicaid or Medicare, reported fair/poor health, or had a comorbid cardiovascular condition were more likely to have a foot examination compared with those who lived in families with incomes below 100% of the FPL, were privately insured, were in excellent/good health, or did not have a cardiovascular comorbid condition. Patients who had incomes greater than 400% of the FPL were more likely to receive a foot examination than patients who lived in families with incomes below 100% of the FPL.

## Discussion

Our study advances the literature on racial/ethnic disparities in quality of care for patients with diabetes. We assessed racial/ethnic disparities in the quality of diabetes care on the basis of receipt of recommended HbA1c tests and foot and eye examinations in the previous year. We hypothesized that racial/ethnic minority patients with diabetes would receive lower quality of care than their white counterparts. Compared with white patients with diabetes, Asian patients with diabetes were less likely to have received at least 2 HbA1c tests and both Asian and Hispanic patients were less likely to have received a foot examination in the past 12 months. Conversely, black patients with diabetes were more likely to have received a foot examination in the past 12 months compared with white patients with diabetes. This finding may be explained by the fact that black patients with diabetes tend to have higher rates of diabetes complications and amputations, and recent guidelines have highlighted the need to carefully monitor these patients as their conditions progress (22). These differences remained significant even after controlling for SES, insurance status, health status, comorbid conditions, and lifestyle behavior variables.

However, our results differ from those found by Lee and colleagues (19), who found no differences in receipt of these measures among racial/ethnic minorities. Their analysis of 2000 MEPS data found no differences among racial/ethnic groups for most of the outcomes in diabetes care management, including respondents who had received an HbA1c test, had their feet checked for sores or irritation, or received an eye examination in the past year. A possible explanation for the different findings may be differences in study design. Contrary to the study conducted by Lee et al (19), we restricted our sample to unique individuals to compute appropriate standard errors in pooled estimations. Additional differences were the use of more recent data sets, the use of special diabetes weights from MEPS, and the use of the balanced repeated replication method variance estimation to account for the full set of survey stratum and primary sampling units, as recommended by MEPS (23). Our results also differ from findings of a study by Trivedi et al that found narrowing of the gap in the quality of diabetes care between whites and blacks (14). However, this study was limited to Medicare beneficiaries in managed care, and the authors did not stratify by other racial/ethnic minority groups such as Hispanics and Asians. The findings by Trivedi et al may not be generalizable to other health systems or to other racial/ethnic groups that may experience greater racial/ethnic disparities in the quality of diabetes care. Conversely, our findings are consistent with those of other studies that used both clinical and community-based data (24-29).

Our study has limitations. First, the data we used were cross-sectional, so causal relationships cannot be established. Second, the dependent variables were self-reported measures of process outcomes of diabetes care. Although we controlled for patients who reported poor or fair health or comorbid cardiovascular conditions, these patients may have visited their providers more often and thus were more likely to receive diabetes tests compared with those who reported excellent or good health and no comorbid cardiovascular conditions. Furthermore, no information on glycemic control among patients with diabetes was available. Asians may have better glycemic control and may have received HbA1c tests and foot and eye examinations less frequently than their white counterparts.

Although the link between racial/ethnic minority status and the quality of care for patients with diabetes is not completely understood, our study suggests that factors such as health insurance status, SES, and self-rated health status are potential antecedents of quality of diabetes care. Therefore, assessing the association between racial/ethnic disparities in the quality of diabetes care and factors such as SES, insurance status, and health behaviors is warranted because these factors are modifiable and can serve as the focus of interventions to reduce racial/ethnic disparities in the quality of diabetes care. Findings from this study may have clinical, public health, public policy, and research implications. Specifically, these results may underscore the importance of providing diversity training to providers to improve the quality of care to patients with diabetes. Furthermore, evidence from this study may play a key role in informing policy makers in their continuous efforts to translate effective research into nationwide practices to eliminate racial/ethnic differences in quality of care, which is relevant in the context of the current health care reform law that seeks to eliminate racial/ethnic disparities. Additional research is needed to fully evaluate the mechanisms and sources of racial/ethnic disparities in the quality of diabetes care.

## Figures and Tables

**Table 1:** Dependent and Independent Variables for Racial/Ethnic Disparities in the Quality of Care for Patients Aged 18 to 64 Years With Diabetes (n = 2,452), 2005-2007 Medical Expenditure Panel Survey^a^

**Characteristics**	Weighted %^b^
**Dependent Variables**	
**Clinical testing**	
Received ≥2 HbA1c tests in past year	83.2
Received foot examination in past year	70.9
Received eye examination in past year	61.1
**Independent Variables**	
**Female sex**	52.1
**Age, y**	
18-24	0.6
25-34	2.7
35-44	9.5
45-54	18.5
55-64	30.5
65-74	19.9
≥75	18.3
**Race/ethnicity**	
Non-Hispanic white	68.2
Black	15.0
Hispanic	13.2
Asian	3.6
**Education**	
Less than a high school degree	33.0
High school degree	51.0
College degree	10.6
Postgraduate degree	5.4
**Income as % of FPL^c^**	
<100	14.1
100-199	21.2
200-400	31.0
>400	33.7
**Insurance status**	
Private insurance	62.7
Public insurance	31.2
Uninsured	6.1
**Health status/conditions**	
Fair/poor health	39.7
Obese^d^	54.7
Comorbid cardiovascular conditions^e^	77.8
**Current smoker**	15.5
**Region**	
Northeast	19.1
Midwest	21.3
South	39.4
West	21.2

Abbreviations: HbA1c, glycated hemoglobin; FPL, federal poverty level.

a Data are pooled for years 2005, 2006, and 2007 of the Household Component of the Medical Expenditure Panel Survey (MEPS). The sample was restricted to unique individuals in each year of the pooled data. There are no repeated observations for the same individual across the different years.

b Percentages weighted to yield a nationally representative sample of US households.

c FPL is the set minimum amount of income that a family needs for food, clothing, transportation, shelter and other necessities and is used to determine eligibility income limits for public assistance programs as some percentage of FPL. FPL varies according to family size and is determined by the US Department of Health and Human Services.

d Reported body mass index of ≥30 kg/m^2^.

e Respondents with any of the following conditions: hypertension, angina, mild or coronary heart attack, stroke, or other form of heart disease.

**Table 2:** Weighted Proportions Receiving 2 HbA1c Tests, Foot Examination, and Eye Examination in the Past Year, by Racial/Ethnic Minority Group, Adult Patients Aged 18 to 64 Years With Diabetes (n = 2,452), 2005-2007 Medical Expenditure Panel Survey^a^

Race/ethnicity	Weighted %^b^ (95% CI)		

	Received ≥2 HbA1c Tests in Past Year	Received Foot Examination in Past Year	Received Eye Examination in Past Year
White	83.0 (80.3-85.8)	71.2 (67.6-74.9)	63.5 (59.2-67.6)
Black	84.5 (79.1-89.9)	76.9 (71.4-82.3)	53.1 (45.1-61.2)
Hispanic	77.8 (71.4-95.6)	63.1 (56.1-70.2)	52.6 (45.7-59.5)
Asian	83.3 (71.1-95.6)	70.2 (53.5-86.9)	75.9 (58.8-92.9)

Abbreviations: CI, confidence interval; HbA1c, glycated hemoglobin.

a Data are pooled for years 2005, 2006, and 2007 of the Household Component of the Medical Expenditure Panel Survey (MEPS). The sample was restricted to unique individuals in each year of the pooled data. There are no repeated observations for the same individual across the different years.

b Percentage is weighted to yield a nationally representative sample of US households.

**Table 3:** Logistic Regression Results for Racial/Ethnic Disparities in the Quality of Care for Patients with Diabetes (n = 2,452), by Quality Indicators, 2005-2007 Medical Expenditure Panel Survey^a^

Independent Variable	Received ≥2 HbA1C Tests in the Past Year		Received Foot Examination in the Past Year		Received Eye Examination in the Past Year	

	AOR (95% CI)	*P* Value	AOR ( 95% CI)	*P* Value	AOR (95% CI)	*P* Value
**Female sex**	1.14 (0.96-1.35)	.13	1.10 (0.90-1.35)	.34	1.14 (0.93-1.40)	.20
**Age, y**						
18-24	1 [Reference]					
25-34	0.51 (0.06-2.36)	.84	0.41 (0.05-2.25)	.82	0.44 (0.06-2.44)	.81
35-44	0.81 (0.10-2.65)	.95	0.66 (0.08-2.63)	.92	0.45 (0.09-2.72)	.82
45-54	0.90 (0.14-3.36)	.97	0.64 (0.13-3.41)	.91	0.58 (0.13-3.43)	.87
55-64	0.97 (0.16-4.08)	.99	0.74 (0.15-4.09)	.94	0.63 (0.16-4.15)	.90
65-74	0.75 (0.12-3.41)	.93	0.79 (0.10-3.45)	.95	0.68 (0.13-3.43)	.91
≥75	0.82 (0.12-3.43)	.95	0.85 (0.11-3.55)	.97	0.85 (0.11-3.40)	.96
**Race/ethnicity**						
Non-Hispanic white	1 [Reference]					
Black	1.10 (0.85-1.42)	.48	1.31 (1.00-1.72)	.05	0.86 (0.63-1.17)	.33
Hispanic	1.13 (0.87-1.46)	.35	0.76 (0.57-1.03)	.08	0.80 (0.60-1.06)	.11
Asian	0.44 (0.20-0.98)	.04	0.35 (0.15-0.81)	.02	0.67 (0.33-1.38)	.27
**Education**						
No high school degree	1 [Reference]					
High school degree	0.81 (0.66-1.00)	.05	0.80 (0.65-0.99)	.04	1.16 (0.91-1.47)	.22
College degree	1.11 (0.77-1.60)	.56	0.97 (0.62-1.50)	.87	1.53 (1.05-2.22)	.03
Graduate degree	0.98 (0.62-1.54)	.93	1.11 (0.61-2.00)	.73	0.92 (0.51-1.64)	.77
**Income as % of FPL^b^**						
<100	1 [Reference]					
100-199	1.15 (0.84-1.57)	.39	1.04 (0.79-1.38)	.77	1.21 (0.89-1.65)	.22
200-400	1.29 (0.91-1.84)	.15	1.00 (0.72-1.40)	.99	1.45 (1.03-2.04)	.04
>400	1.32 (0.99-1.76)	.06	1.36 (1.00-1.85)	.05	1.81 (1.18-2.77)	.01
**Insurance status**						
Private insurance	1 [Reference]					
Public insurance	1.02 (0.83-1.24)	.86	1.33 (1.05-1.68)	.02	0.94 (0.72-1.21)	.61
Uninsured	0.91 (0.65-1.26)	.55	0.78 (0.53-1.15)	.21	0.48 (0.32-0.72)	.001
**Health status/conditions**						
Fair/poor health	1.22 (0.98-1.53)	.08	1.37 (1.10-1.71)	.01	0.96 (0.77-1.19)	.70
Obese	1.03 (0.86-1.24)	.74	1.05 (0.87-1.28)	.60	1.03 (0.80-1.33)	.80
Cardiovascular comorbidity	1.34 (1.04-1.71)	.02	1.39 (1.03-1.87)	.03	1.24 (0.88-1.74)	.21
**Current smoker**	1.01 (0.78-1.31)	.92	1.08 (0.81-1.43)	.61	0.72 (0.55-0.93)	.01
**Region**						
Northeast	1 [Reference]					
Midwest	1.01 (0.69-1.48)	.97	0.61 (0.43-0.87)	.01	0.96 (0.69-1.33)	.81
South	0.91 (0.67-1.24)	.56	0.69 (0.54-0.89)	.004	0.79 (0.56-1.13)	.19
West	0.82 (0.56-1.20)	.30	0.78 (0.60-1.01)	.06	0.84 (0.62-1.14)	.26

Abbreviations: HbA1c, glycated hemoglobin; AOR, adjusted odds ratio; CI, confidence interval; FPL, federal poverty level.

a Data are pooled for the 2005, 2006, and 2007 waves of the household component of the Medical Expenditure Panel Survey. The sample was restricted to unique individuals in each of the rounds of the pooled data. There are no repeated observations for the same individual across the different rounds of the year.

b The set minimum amount of income that a family needs for food, clothing, transportation, shelter and other necessities and is used to determine eligibility income limits for public assistance programs as some percentage of FPL. FPL varies according to family size and is determined by the US Department of Health and Human Services.
